# Psychometric Validity of the Visual Function Index in Leber Hereditary Optic Neuropathy

**DOI:** 10.1167/tvst.12.1.23

**Published:** 2023-01-20

**Authors:** Benson S. Chen, Patrick Yu-Wai-Man, Mike Horton

**Affiliations:** https://orcid.org/0000-0001-8214-0186; https://orcid.org/0000-0002-6675-7335; 1 John van Geest Centre for Brain Repair and MRC Mitochondrial Biology Unit, Department of Clinical Neurosciences, University of Cambridge, Cambridge, UK; 2 Cambridge Eye Unit, Addenbrooke's Hospital, Cambridge University Hospitals, Cambridge, UK; 3 Moorfields Eye Hospital NHS Foundation Trust, London, UK; 4 Institute of Ophthalmology, University College London, London, UK; 5 Psychometric Laboratory for Health Sciences, University of Leeds, UK

**Keywords:** Leber hereditary optic neuropathy, LHON, quality of life, VF-14, visual function index, rasch analysis

## Abstract

**Purpose:**

The purpose of this study was to determine the psychometric validity of the Visual Function Index (VF-14) for use by patients with Leber hereditary optic neuropathy (LHON).

**Methods:**

Rasch analysis was conducted in two stages using data for 196 individuals (74.5% male) carrying one of the three primary LHON mutations and affected by vision loss. In stage 1, scale unidimensionality, scale-to-sample targeting, response category threshold ordering, item fit statistics, local dependency, and reliability were assessed. In stage 2, iterative post-hoc revisions of the VF-14 structure (VF-14R) were applied and psychometrically re-evaluated.

**Results:**

Issues identified with the VF-14 included disordered response thresholds (12/14 items), local dependency (10/91 pairwise dependencies), and evidence of multidimensionality. However, the distribution of person estimates and item thresholds were fairly well matched, only one item showed misfit to the Rasch model, and there was good reliability (Person Separation Index 0.84). Rasch-informed VF-14 revisions included removing both driving items and the misfitting sports item, rescoring response options across all items by merging two response categories, and accounting for the dependency between two reading items. The VF-14R demonstrated improved psychometric validity.

**Conclusions:**

Clinicians and researchers using the VF-14 with LHON patients should be aware of its limitations. Compared to the original version, the proposed Rasch-based structure of the VF-14R appears to offer improved psychometric performance and interpretation of vision-related activity limitation.

**Translational Relevance:**

The original version of the VF-14 exhibits several limitations that undermines its psychometric validity as a patient-reported outcome measure for patients with LHON.

## Introduction

Impact on vision-related quality of life is a frequently desired outcome measure in therapeutic clinical trials for eye-related conditions, where it is recognized that traditional metrics of visual function do not comprehensively capture the patient experience.^1^^,^^2^ This has led to an explosion of patient-reported outcome measures (PROMs) for eye-related conditions, enabling patients to provide their views about a range of issues including visual and general symptoms, social and emotional well-being, activity limitation, mobility, and health concerns, without interpretation by a clinician.^1^

Measuring the impact of new treatments in rare diseases is especially challenging, as there is a lack of disease-specific PROMs that comprehensively capture the range of issues that patients with rare diseases experience.^3^^,^^4^ Some PROMs, such as the 14-item Visual Function Index (VF-14), originally designed to measure the functional impairment of cataracts,^5^ have been used by patients with other eye-related conditions, including Leber hereditary optic neuropathy (LHON).^6^^–^^8^

LHON is a maternally inherited mitochondrial disease that manifests with subacute, sequential, bilateral vision loss.^9^ The estimated prevalence of LHON in Northern Europe is approximately one in 30,000 to 50,000. Patients typically develop vision loss between the ages of 15 and 35 years old. Vision loss in LHON is devasting. The characteristic visual defect in LHON is a dense central or cecocentral scotoma. In most patients, visual acuity ranges from counting finger to perception of light, with little recovery. Three primary point mutations in mitochondrial DNA (m.3460G>A, m.11778G>A, and m.14484T>C) are causative of LHON in approximately 90% of those affected. Treatments for LHON are limited. However, several Phase III gene therapy clinical trials have been recently completed.^10^^–^^12^

The VF-14 has been used to assess the quality of life of individuals with LHON in at least three studies, including a multicenter, double-blind, randomized, placebo-controlled trial (RHODOS), where it was used as a trial endpoint for health-related quality of life.^7^^,^^8^^,^^13^ Although the validity of the VF-14 has been established for other conditions,^14^^,^^15^ the validity of VF-14 has never been assessed when used by individuals affected by LHON. The aim of this study was to determine the psychometric validity of the VF-14 for use by individuals affected by LHON.

## Methods

### Participants

Participants were recruited from clinical databases maintained by the Mitochondrial Research Group, Newcastle University (Newcastle-upon-Tyne, United Kingdom), Erasmus Medical Center (Rotterdam, the Netherlands), and Department of Neurology, Friedrich-Baur-Institute (Munich, Germany). Participants were included if they were confirmed to have one of the three primary LHON mutations and were affected by vision loss because of LHON. Asymptomatic carriers and individuals with vision loss from other causes or LHON-plus syndrome were excluded. Details regarding recruitment have been described in a previous study.^8^ The study received ethical approval from the relevant Institutional Review Board of the three participating institutions and complied with the Declaration of Helsinki.

### VF-14

The VF-14 was administered to study participants by three investigators via telephone. Participants were asked to give answers that incorporated the use of reading glasses, but not visual aids such as magnifiers.

The VF-14 asks respondents to rate their ability in performing 14 vision-dependent activities in everyday life and the degree of difficulty for each activity.^5^ Five response categories are provided for each item: 0 (“unable to do”), 1 (“a great deal of difficulty”). 2 (“moderate difficulty”), 3 (“a little difficulty”), and 4 (“no difficulty”). If an activity is unable to be performed for other reasons, the item is marked “not applicable.” In the original VF-14, an average score was generated from all answered items and multiplied by 25 to give a scale ranging from 0 (worst level) to 100 (best level) of visual function.^13^

### Statistical Analysis

Statistical analysis of demographic data was performed with R: A language and environment for statistical computing (version 4.0.2, R Foundation for Statistical Computing, http://www.R-project.org). The psychometric validity of the VF-14 was assessed by Rasch analysis using RUMM2030 Plus (RUMM Laboratory, Perth, Australia).

Rasch measurement theory is a modern psychometric method that fits data to the unidimensional Rasch model. When the assumptions of the Rasch model are satisfied, this allows for the transformation of raw ordinal scores into interval-level measurement expressed as a log odds unit (logit), thereby permitting the use of parametric statistical techniques.^16^^,^^17^ The Rasch model considers the performance of a person on an item to be a product of the difference between a person effect or “ability” (the level of the underlying trait being measured, e.g., visual function) and an item effect (item difficulty). Unlike other models of item response theory, Rasch measurement theory is principally concerned with how the data fits the Rasch model by comparing the pattern of observed responses with the model expectation, and highlighting measurement anomalies within a set of items. Rasch analysis provides insight into the psychometric properties of the scale (Table 1). Item sets can be calibrated with the Rasch model, and when measurement anomalies are identified, post-hoc adjustments can be made to items to account for certain issues, allowing them to be “re-engineered” to fit the Rasch model. This process creates valid interval-level person measurements to which parametric statistics can be applied.

**Table 1.** Definitions of Performance Measures That Can be Determined by Rasch Analysis^30^^–^^35^

Performance Measure	Definition
Response category threshold ordering	Category thresholds are the point at which a respondent is equally likely to select two adjacent response categories. Response category thresholds are “ordered” when the transition point from one response level to the next increases in an ascending order, concordant with increasing levels of the underlying construct or trait being measured. This can be assessed by inspecting the category probability curves to determine the ordering of the response probabilities. “Disordered” response category thresholds can indicate too many response categories or response category relevancy/redundancy issues.
Item fit statistics	Fit statistics measure how closely the observed data deviates from expected estimates. This is determined by standardizing the individual item and person fit residuals to approximate a Z-score, where Z-scores between ±2.5 indicate an adequate fit to the Rasch model. χ^2^ statistics are used to determine how well the data meets the requirement of the Rasch model, with a probability value of *P <* 0.05 (with adjustment for multiple testing applied at the item level) indicative of deviation from the Rasch model. Individual item fit can be represented graphically against the Rasch model's item characteristic curve, providing additional information of an item's ability to discriminate between different groups.
Local dependency	Local dependency occurs when items are linked in a manner that is over and above what is explained by the underlying trait. Local dependency within pairwise items can be assessed by analyzing the correlation of item residuals. If the correlation between two items is >0.2 above the average correlation, this indicates that the pairwise items are locally dependent. If the indicated dependency is also supported conceptually, then items that correlate together may be grouped (into a “super-item”) or a single item removed in order to account for the dependency between the items.
Scale-to-sample targeting	Ability of the scale to measure different levels of the underlying construct or trait, across the full range of abilities of the group being assessed. Poorly targeted scales are limited by floor or ceiling effects, where the scale is unable to differentiate between respondents with very high or very poor ability. Poorly targeted scales also display an uneven spread of items across the full range of abilities of the group being assessed, indicating that additional items are required.
Reliability	Ability of the scale to distinguish between different levels of the underlying construct or trait, often assessed using the PSI, which is analogous to Cronbach's alpha, a measure of internal consistency. PSI scores range between 0 and 1. A PSI value of 0.7 is the minimum accepted level and represents the ability of the scale to distinguish two distinct groups of person ability, whereas a PSI value of 0.9 represents the ability to distinguish four distinct groups of person ability.
Unidimensionality	A property of a scale, indicating that it is measuring a single underlying construct or trait. Unidimensionality is assessed using a series of paired t-tests. Where unidimensionality is assumed, and there is no conceptual or subscale separation of items, the pattern of factor loadings on the first component of a Principal Component Analysis of the residuals allows subsets of “positive” and “negative” loading items to be identified. In the series of *t*-tests, a paired *t*-test is used for each person to determine whether person estimates derived from these two subsets of items statistically differ. Unidimensionality is supported if the percentage of significant *t*-tests (at *P* < 0.05) is less than 5%.

Rasch analysis of the VF-14 was conducted in two stages. In stage 1, response category threshold ordering, item fit statistics, local dependency, scale-to-sample targeting, scale reliability, and scale unidimensionality were assessed. In stage 2, iterative post-hoc revisions of the VF-14 structure (VF-14R) were applied and psychometrically re-evaluated.

## Results

One hundred ninety-six individuals (74.5% male) with LHON completed the VF-14. The median age of respondents was 41 years (interquartile range, 29–55 years), with median disease duration of nine years (interquartile range, 3–25 years). Most respondents (67.3%) harbored the m.11778G>A LHON mutation, followed by m.3460G>A mutation (17.9%) and the m.14484T>C mutation (14.8%).

**Figure 1.**
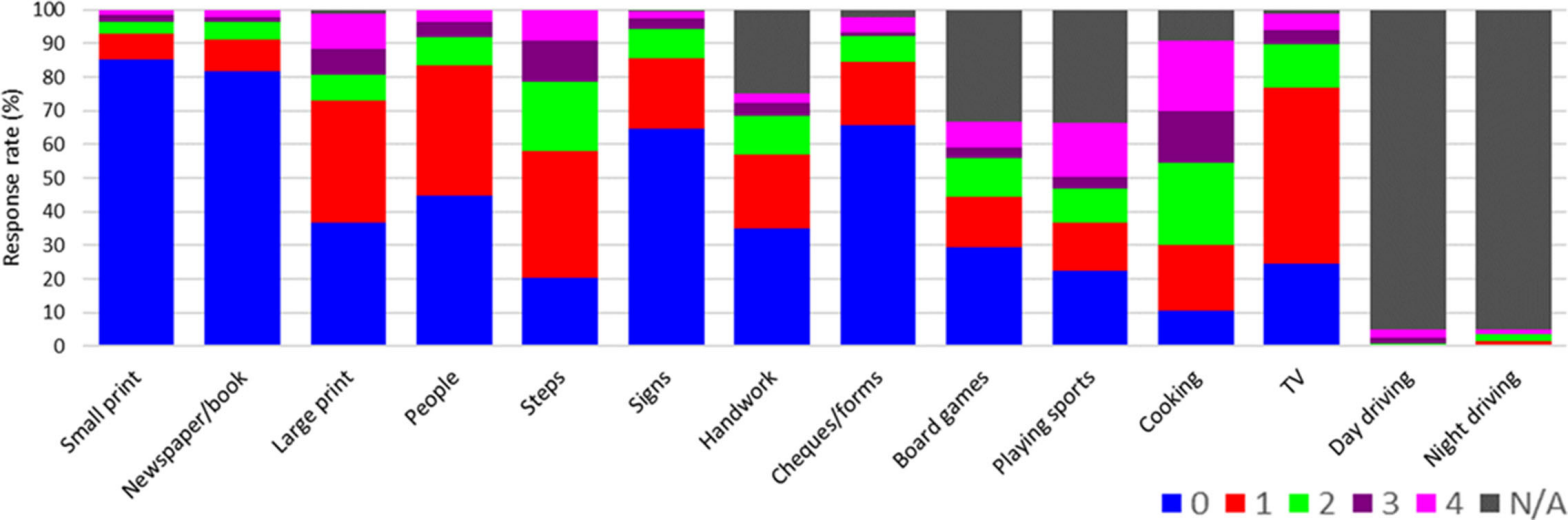
Distribution of responses to items of the VF-14 by 196 individuals with LHON LHON: Leber hereditary optic neuropathy; VF-14: Visual Function Index. Response categories: 0 (“unable to do”); 1 (“a great deal of difficulty”); 2 (“moderate difficulty”); 3 (“a little difficulty”); 4 (“no difficulty”); and N/A (“not applicable” – i.e., unable to do for reasons other than vision).

The median VF-14 score was 17.1 (interquartile range, 10.4–31.3), using the scoring system out of 100 recommended by the developers. Only 10 respondents provided a complete response for all 14 items without a “not applicable” response. Four items had complete responses (“reading small print,” “reading newspaper/book,” “recognizing people,” and “seeing steps”). Of the 10 items that had incomplete responses, both driving items (“driving during the day” and “driving at night”) had the lowest response rate (5.1% for both items), indicating that most respondents did not drive, either because they had stopped driving for reasons other than their vision or had never learned to drive for whatever reason. Other items with incomplete responses included “participation in sports” (33.5% incomplete responses), “playing games” (33.0%), “doing fine handwork” (24.9%), and “cooking” (9.1%). The distribution of responses across all 14 items are summarized in Figure 1.

### Stage 1

**Figure 2.**
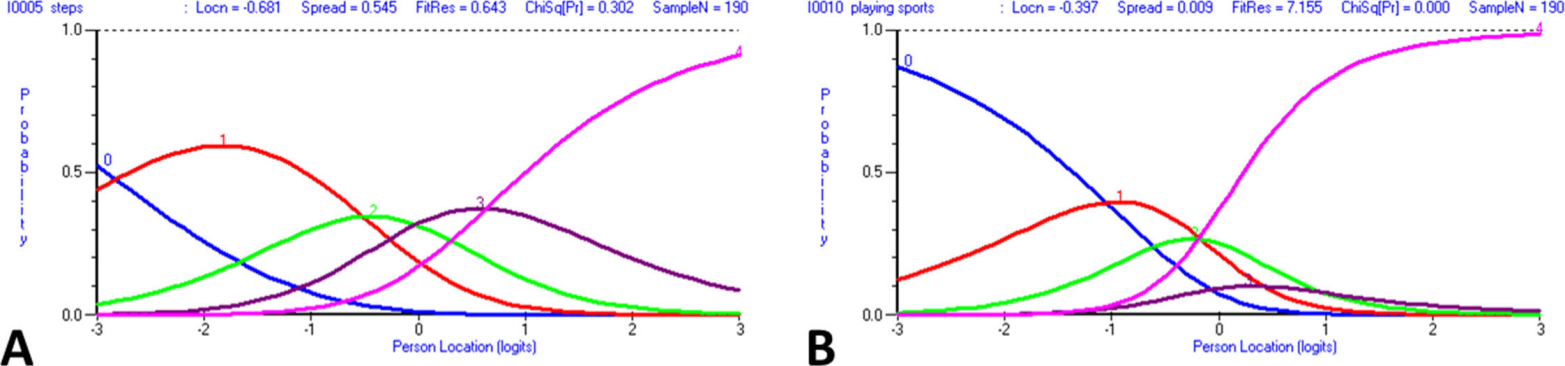
Response category probability curves. (A) The probability curves for the “seeing steps” item are ordered, with the response category thresholds (i.e., the junction between each response category) ascending in an ordered fashion along the continuum of person location. (B) The probability curves for the “participation in sports” item do not ascend in an ordered fashion. The junction between response category 0 (“unable to do”) and 1 (“a great deal of difficulty”) intersects at a person location of −1.05 logits, whereas the junction between response category 3 (“a little difficulty”) and 4 (“no difficulty”) intersects at a lower person location (−1.4 logits). Note the non-functioning of response category 3, in particular.

**Figure 3.**
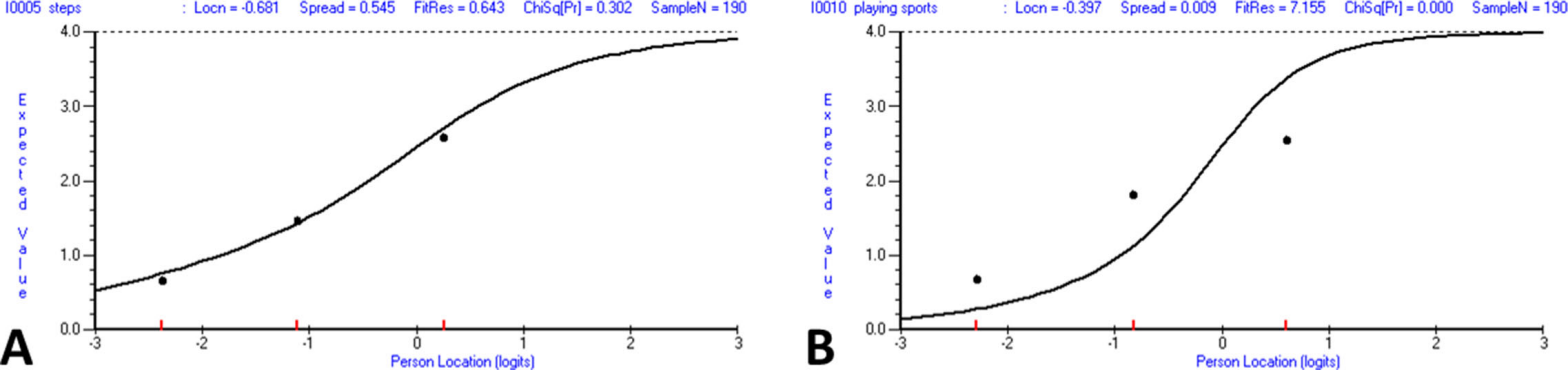
Item characteristic curves. (A) The Item characteristic curve for the “seeing steps” item shows excellent fit to the Rasch model, with a nonsignificant fit residual statistic well within ±2.5. (B) The item characteristic curve for the “participation in sports” item shows poor fit to the Rasch model, with an under-discrimination apparent. The fit residual statistic is 7.2 and χ^2^ probability is highly significant.

Initial fit of the VF-14 to the Rasch model was poor (χ^2^(28) = 96.67, *P* < 0.01). Only two items (“seeing steps” and “doing fine handwork”) showed ordered response category thresholds. For the items that showed disordered response category thresholds, there was frequent nonfunctioning of the middle response categories, especially the “a little difficulty” category (Fig. 2). When examined for individual item fit (Table 2), only one item (“participation in sports”) showed misfit to the Rasch model (Fig. 3), and appeared to be an under-discriminating item.

**Table 2.** Item Fit Statistics and Parameter Values for Items of the Original Version of the VF-14

No.	Item	Location (Logits)	Standard Error	Fit Residual	χ^2^ Probability
1	Small print	1.419	0.133	−0.782	0.129
2	Newspaper/book	1.332	0.128	−2.213	<0.05
3	Large print	−0.329	0.086	−1.438	<0.05
4	People	0.391	0.101	−0.483	<0.05
5	Steps	−0.681	0.089	0.643	0.302
6	Signs	0.871	0.108	−2.313	0.112
7	Handwork	0.571	0.111	1.370	0.179
8	Cheques/forms	0.661	0.105	−1.67	0.132
9	Board games	0.034	0.104	−0.429	0.051
10	Playing sports	−0.397	0.095	7.155	<0.05
11	Cooking	−1.433	0.086	1.211	0.466
12	Television	−0.038	0.103	−0.095	0.168
13	Day driving	−1.300	0.356	−0.686	0.264
14	Night driving	−1.102	0.342	−0.515	0.792

Testing for local item dependency identified 10/91 pairwise dependencies. The “reading newspaper/book” item exhibited local dependence with three other items: “reading small print,” “reading large print,” and “reading signs.” The remaining six pairwise dependencies involved both driving items, paired with conceptually unrelated items including “cooking.”

**Figure 4.**
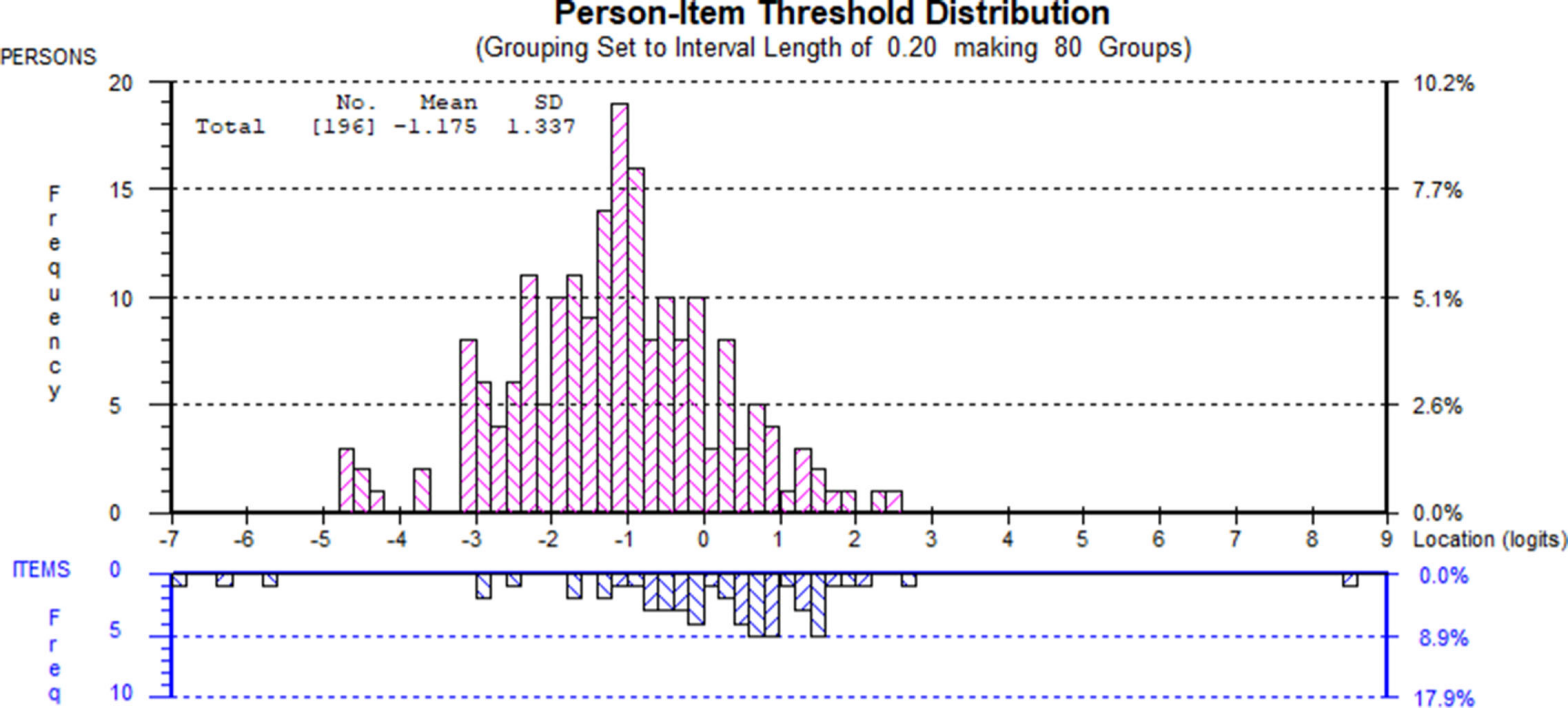
Person-item threshold distribution for the original version of VF-14. The *blocks* in the upper part of the chart represent groups of respondents across a continuum of visual ability, represented by the x-axis (“Location” in logits). The blocks in the lower part of the chart represent the location of item thresholds and their distribution. The VF-14 has reasonable scale-to-sample targeting, with a mean person location of −1.18 logits, compared to a mean item location of 0 logits. There is a good spread of item thresholds across the continuum of respondents” visual abilities, but the VF-14 appears to target those with better visual ability and less vision-related activity limitation. There are less items that target those with lower visual ability and more vision-related activity limitation.

Figure 4 shows the person-item threshold distribution of individuals completing the VF-14. There was reasonable spread of estimated person ability and item difficulty along the logit continuum. The mean (± standard deviation) of person ability was −1.18 ± 1.34 logits, with the mean location of item difficulty centralized at 0 ± 0.93 logits, indicating that respondents were, on average, at a lower level of visual functionality than the average level represented by the items. This means that, generally, the people within this sample were less likely to affirm more difficult items (i.e., items that require better visual function such as reading small print). There was no ceiling effect. However, there was evidence of a minor floor effect, with six respondents attaining the minimum raw score of 0. Despite this, the Person Separation Index (PSI) was 0.84, indicating good reliability. The VF-14 in its original format exhibited evidence of multidimensionality with 12.8% of paired *t*-tests significant (at *P* < 0.05), indicating that that the VF-14 appeared to be measuring more than one underlying construct.

### Stage 2

Post-hoc revisions to the structure of the VF-14 (VF-14R) were made in response to the anomalies identified in stage 1. Both driving items were removed in response to the very low response rates. The “participation in sports” item was also removed due to significant misfit to the Rasch model. In order to correct the disordered response thresholds, a post-hoc rescore was applied across all items where the response categories “moderate difficulty” and “a little difficulty” were merged. Additionally, the “reading small print” and “reading newspaper/book” items were merged into one “super item” to account for the local dependency exhibited between them, because both items were conceptually similar.

After these post-hoc adjustments, the VF-14R was psychometrically re-evaluated, and satisfactory fit to the Rasch model was demonstrated (χ^2^(20) = 31.23, *P* = 0.052). All items displayed ordered response categories, and none of the items were misfitting. Only 1/45 item pair dependencies were identified. However, the item pair (“cooking” and “playing games”) dependency was borderline (Q3 residual correlation value 0.091; criterion for local independence <0.089), and the conceptual link between these items is less clear. The Person-Item Threshold Distribution of the VF-14R continued to show reasonable spread of estimated person ability and item difficulty along the logit continuum, with a minor floor effect. There was good reliability with a PSI of 0.852. Unidimensionality of the VF-14R was confirmed (2.8% of paired *t*-tests significant at the *P* < 0.05 level).

## Discussion

Clinicians and researchers working with individuals with LHON should be aware of the limitations of the VF-14 in its original format. When used by individuals with LHON, the original version of the VF-14 exhibits several shortcomings that impact its psychometric validity. Key issues identified in this study included problems with the structure of response categories and evidence of scale multi-dimensionality. Respondents with LHON in this study tended to endorse the categories “unable to do” and “a great deal of difficulty” for most items in the VF-14, resulting in left-skew of the distribution of person scores and a minor floor effect, i.e. a lower limit to the data values that the VF-14 can reliably specify. The response category “a little difficulty” was seemingly underused across almost all items, with respondents being always more likely to respond in one of the adjacent response categories. These problems likely arise from differences in the visual abilities of individuals with LHON compared to those with cataracts, for whom the VF-14 was originally designed for. The effect of cataracts on vision is widely variable, as cataracts may be unilateral or bilateral and can vary widely in size, morphology, and degree of lens opacification. Whereas vision loss in LHON is generally bilateral and severe, with the majority of patients with LHON significantly visually impaired with visual acuity of 6/60 or less.^18^^,^^19^

In its original format, the VF-14 also demonstrated evidence of multidimensionality. Measurement scales that are multidimensional are problematic as they indicate that different items are measuring different traits, thereby reducing the reliability of the scale.^16^ Apparent multidimensionality can also be indicated where local dependency is present within a scale.^20^ We found that by removing the “participation in sports” item and both driving items, along with accounting for the dependency between the “reading small print” and “reading newspaper/book” items, the VF-14R became a unidimensional scale. The “participation in sports” item showed misfit to the Rasch model and an underdiscrimination for respondents with differing levels of visual ability, indicating that it was performing poorly as a measure of vision-related activity limitation. This is not surprising as the amount of vision required for sports participation depends on the type of sport, of which the VF-14 lists “bowling, handball, tennis, and golf” as examples. In contrast, driving is very dependent on visual ability. However, assessing difficulty driving in a vision-related PROM with multiple response categories is problematic, as responses will typically favor those with good vision that meet the legal requirement for driving and, hence, responses in the “no difficulty” and “a little difficulty” categories.

The performance of the VF-14 was also impacted by incomplete responses marked with “not applicable.” Nearly one quarter or more of respondents did not respond to at least one of five items (“participation in sports,” “playing games,” “doing fine handwork,” “driving during the day,” and “driving at night”). Differences in the demographic background of individuals with LHON compared to individuals with cataracts, likely explain the incomplete responses. The peak age of onset of vision loss in LHON is between 15 and 35 years, with a subset of patients under the age of 12 years developing a form of childhood-onset LHON.^21^ The examples used for sports (“bowling, handball, tennis, and golf”), games (“bingo, dominos, card games, and mahjong”), and fine handwork (“sewing, knitting, crocheting, and carpentry”) are more relevant for older individuals with cataracts compared to younger individuals with LHON. Missing responses, particularly for the driving items, lead to larger standard errors (Table 2) and reduced precision of estimates. Incomplete responses for the driving items may be related to two factors. First, individuals with LHON, especially those with childhood-onset LHON, may have never had the opportunity to learn to drive because of legal requirements regarding age. Second, respondents in this study were recruited from European countries including Germany, the Netherlands, and the United Kingdom, where there is more widespread public transportation use compared to the United States, where the VF-14 was originally developed. Although both driving items were excluded from the VF-14R, they can still be retained as nonscoring items because they provide useful clinical information that serve as a benchmark for the level of visual function legally required to drive.

For PROMS to be effective as an outcome measure in clinical trials, they have to capture the disease characteristics that matter to the patient and must be reliable and valid.^4^ The VF-14 was previously used in the RHODOS trial to measure change in health-related quality of life.^13^ Only small changes in the VF-14 score were observed over the 24-week study period.^22^ A small nonsignificant treatment effect was detected between the idebenone- and placebo-treated groups. As we have demonstrated in this study, there are issues with the validity of the VF-14 that limit its usefulness. Furthermore, the VF-14 only measures vision-related activity limitation and does not specifically address other domains of quality of life that may be important to individuals with LHON.^23^^,^^24^ In this study we have demonstrated that the psychometric properties of the VF-14 can be improved by re-engineering the response categories and removing items that don’t fully contribute towards the total score. Additional studies could be performed to determine the impact of idebenone on vision-related activity limitation using the VF-14R we have proposed. However, post-hoc revisions to the VF-14 does not address the content of the PROM, which would require additional items to function as a measure of health-related quality of life.^23^ For these reasons, we have not provided equating scales to convert the original VF-14 to the modified VF-14R score.

A weakness of the VF-14 in its original format is the conversion of raw scores for individual items into an average score out of 100. This assumes that each item contributes equally to the final score, and that the interval between each response category is uniform across all the categories and for all items. A key strength of this study was the use of Rasch analysis to overcome this limitation by calculating item difficulty in relation to person ability and adjusting the overall scores accordingly, allowing comparison of measures and interpretation of changes in scores when the logit scores are used. Previous studies have found that Rasch-informed revisions to the VF-14 are more sensitive to change than the original VF-14, when used in longitudinal studies of patients with cataracts who undergo surgery.^25^ Another strength of Rasch analysis is its robustness to incomplete data, with all available data being used within the analysis process.^26^ This is a substantial advantage over Classical Test Theory, a traditional psychometric approach, where item-level missing data can bias test results.^27^

Limitations of this study relate to the study participants. Individuals with LHON were recruited from large university clinics in three European countries. Patients seen in these clinics may have more complex disease or severe disease because of referral bias. Except for one respondent of Asian descent, all respondents were white. The experiences of these respondents residing mainly in Europe may differ from those elsewhere in the world (e.g., in the United States).^28^^,^^29^ Consistent with the epidemiology of LHON, over 70% of respondents were male, and nearly 70% harbored the m.11778G>A LHON mutation in this study. Additional studies would be required to determine whether any items of the VF-14 exhibited differential item functioning, particularly for female participants or those who carried other LHON mutations.

In summary, the VF-14 in its original format exhibits several limitations that undermines its psychometric validity as a PROM for assessing vision-related activity limitation in individuals with LHON. These limitations likely stem from differences in the clinical and demographic characteristics of individuals with LHON compared to those with cataracts, leading to problems with disordered response category thresholds, item misfit, and scale multidimensionality. Rasch informed post-hoc revisions of the VF-14 can improve the psychometric validity of the PROM. However, these revisions do not overcome issues relating to PROM content. Future studies should evaluate the sensitivity of other established scales for conditions that have similar clinical and demographic characteristics to individuals with LHON, such as people with low vision, or focus on the development of a LHON-specific PROM that captures the disease characteristics that matter most to LHON patients.
